# Quality of life in infants and children with atopic dermatitis: Addressing issues of differential item functioning across countries in multinational clinical trials

**DOI:** 10.1186/1477-7525-5-45

**Published:** 2007-07-27

**Authors:** Stephen P McKenna, Lynda C Doward, David M Meads, Alan Tennant, Gemma Lawton, Jens Grueger

**Affiliations:** 1 Department of Psychology, University of Central Lancashire, Preston, UK; 2 Galen Research, Manchester, UK; 3 Academic Unit of Musculoskeletal & Rehabilitation Medicine, University of Leeds, Leeds, UK; 4 Pricing and Health Economics, Novartis Pharma AG, Basel, Switzerland

## Abstract

**Background:**

A previous study had identified 45 items assessing the impact of atopic dermatitis (AD) on the whole family. From these it was intended to develop two separate scales, one assessing impact on carers and the other determining the effect on the child.

**Methods:**

The 45 items were included in three clinical trials designed to test the efficacy of a new topical treatment (pimecrolimus, Elidel cream 1%) in the treatment of AD in infants and children and in validation studies in the UK, US, Germany, France and the Netherlands. Rasch analyses were undertaken to determine whether an internationally valid, unidimensional scale could be developed that would inform on the direct impact of AD on the child.

**Results:**

Rasch analyses applied to the data from the trials indicated that the draft measure consisted of two scales, one assessing the QoL of the carer and the other (consisting of 12 items) measuring the impact of AD on the child. Three of the 12 potential items failed to fit the measurement model in Europe and five in the US. In addition, four items exhibiting differential item functioning (DIF) by country were identified. After removing the misfitting items and controlling for DIF it was possible to derive a scale; The Childhood Impact of Atopic Dermatitis (CIAD) with good item fit for each trial analysis. Analysis of the validation data from each of the different countries confirmed that the CIAD had adequate internal consistency, reproducibility and construct validity.

The CIAD demonstrated the benefits of treatment with Elidel over placebo in the European trial. A similar (non-significant) trend was found for the US trials.

**Conclusion:**

The study represents a novel method of dealing with the problem of DIF associated with different cultures. Such problems are likely to arise in any multinational study involving patient-reported outcome measures, as items in the scales are likely to be valued differently in different cultures. However, where all items in a scale fit both a single theoretical construct and the Rasch measurement model, it is feasible to conceive of outcome measures with a different set of items in each language.

## Background

Paediatric atopic dermatitis (AD) is a common skin condition affecting 12–15% of children in early childhood [1]. There is a wide variation in the experience of symptoms which, although mild for a majority of children, are subject to unpredictable exacerbations. The disease can have considerable impact on children and their development [2,3] and family life may also be disrupted by the condition [4-6].

A major problem in the assessment of the impact of AD on very young children is their inability to provide the necessary information. Consequently, an alternative approach was taken by McKenna and colleagues [7] who undertook a study designed to look at the impact of the disease and its treatment on the family as a whole. Qualitative interviews were conducted with the principal carers of affected children to identify items for inclusion in a QoL questionnaire. Forty-five potential items were identified covering issues of relevance to the affected child and to his or her siblings and parents. This item set was included in three clinical trials (two in the US and one in Europe) designed to test the efficacy of a new topical treatment for AD (pimecrolimus, Elidel^® ^1% cream) in the treatment of AD in children.

In parallel with the trials, validation studies of the new instrument were conducted in the UK, US, France, Germany and the Netherlands. Rasch analyses applied to the data from these validation studies indicated that the 45 draft items split into two separate scales. The first scale included items such as '*I feel I have no time to relax*' and '*I worry about the way he looks*', that were concerned with the parent's QoL. This was named the Parents' Index of Quality of Life – Atopic Dermatitis (PIQoL-AD). This scale was shown to be unidimensional and to have good internal consistency, test-retest reliability and construct validity [7]. The measure proved effective in the trials in establishing the QoL benefits for parents when Elidel cream is compared to conventional therapy in the treatment of children with AD [8].

A second potential scale was identified that included potential items such as '*He is very moody*' and '*He is very demanding*' that were concerned with the direct impact of AD on the affected child (as reported by the parent) [7]. It is important to note that these items relate to issues that are observable rather than covering social or emotional impacts of which the parent may not be aware.

The present paper reports on analyses undertaken to determine whether the twelve child-specific items could be combined to form a unidimensional scale. If a valid scale could be identified from these items it was intended to call it the CIAD (Childhood Impact of Atopic Dermatitis).

During the development of the PIQoL-AD particular attention had been paid to the production of language versions that would allow trial data to be combined from different countries. It is generally assumed that a measure, where carefully adapted, will be equivalent across language versions, allowing data to be combined validly. Unfortunately, evidence derived from applying the Rasch model to data from different language versions of the same measure does not support this assumption. For example, experience with the Recurrent Genital Herpes Quality of Life questionnaire (RGHQoL) in recurrent genital herpes indicates that the item, *Herpes makes me feel dirty *is valued differently in the US and Germany [9]. Application of the measure suggests that a positive response to this item indicates greater impairment of QoL in the US than in Germany. Such differences are likely to be related to cultural issues that are beyond the scope of the traditional method of adapting questionnaires. In the case of the PIQoL-AD, the method of simultaneous development of different language versions ensured that such cultural differences were kept to a minimum. This was achieved by ensuring that items included in the measure had been raised as important issues in each country where patient interviews were completed and by extensive testing of the draft questionnaire with samples of patients in each country for which a version was developed. Items that exhibited differential item functioning between countries were also removed from the measure.

The present analyses were also designed to determine whether the CIAD can be applied validly in all five countries in which validation studies were conducted.

## Methods

For the present analyses only the 12 items directly relevant to the impact of AD on the affected child were considered. A 'True'/'Not true' response format was employed in these items. All the items used are gender specific with parallel versions of the measure being available for parents of boys and parents of girls.

Three sets of analysis were conducted:

1. Rasch analyses were conducted on the trial data to identify whether a unidimensional scale could be identified that assessed the direct impact of AD on the child.

2. Analyses of the country-specific validation studies were conducted to test the psychometric properties of the identified scale.

3. Finally, the trial data were re-analysed to determine the direct effect of Elidel on the child as assessed by the CAID.

### 1. Trial analyses to identify unidimensional CIAD scale

Data were collected from three double-blind, multi-center, randomized, parallel-group studies in which patients aged 3 months to 18 years were recruited and randomized to either pimecrolimus cream 1% or vehicle cream (in addition to emollients as skincare and medium potency topical steroids for treatment of severe flares) in a 2:1 ratio, respectively. CIAD data were available from parents of children aged up to eight years in the UK, France, Germany, the Netherlands and USA.

In the European trial participants were followed for 12 months with assessments made at baseline, 6 weeks, 6 months and 12 months (Times 1 to 4). Two hundred and eight participants entered the study, of whom, 140 (67.3%) were in the Elidel group and 68 (32.7%) in the placebo (vehicle) group.

In the two US trials, assessments were made at baseline, 6 weeks and 6 months (Times 1 to 3), when the studies were terminated. For the present analyses, the data from these two studies are combined and referred to as the US trial. Of the 261 participants who entered the study, 243 provided a baseline assessment. Of these, 159 (65.4%) were in the Elidel group and 84 (34.6%) in the placebo group.

#### Rasch analyses

Rasch analyses were conducted on the data from the clinical trials in order to determine whether unidimensional CIAD scales could be identified. Separate analyses were run for the US trials and the European trial.

The Rasch model [10] is an unidimensional model that, in educational settings makes the assertion: that the easier an item (question) is the more likely it will be passed, and that the more able a person, the more likely he or she will be to pass an item. It assumes that the probability of a respondent passing a particular item is a logistic function of the relative distance between the item location and the respondent location on a linear scale. In other words, the probability that a person will affirm an item is a logistic function of the difference between the person's ability [*θ*] and the difficulty of the item [b] (i.e. the ability required to affirm item i), and only a function of that difference.

pni=e(θn−bi)1+e(θn−bi)
 MathType@MTEF@5@5@+=feaafiart1ev1aaatCvAUfKttLearuWrP9MDH5MBPbIqV92AaeXatLxBI9gBaebbnrfifHhDYfgasaacH8akY=wiFfYdH8Gipec8Eeeu0xXdbba9frFj0=OqFfea0dXdd9vqai=hGuQ8kuc9pgc9s8qqaq=dirpe0xb9q8qiLsFr0=vr0=vr0dc8meaabaqaciaacaGaaeqabaqabeGadaaakeaacqWGWbaCdaWgaaWcbaGaemOBa4MaemyAaKgabeaakiabg2da9maalaaabaGaemyzau2aaWbaaSqabeaacqGGOaakiiGacqWF4oqCcqWGUbGBcqGHsislcqWGIbGydaWgaaadbaGaemyAaKgabeaaliabcMcaPaaaaOqaaiabigdaXiabgUcaRiabdwgaLnaaCaaaleqabaGaeiikaGIae8hUdeNaemOBa4MaeyOeI0IaemOyai2aaSbaaWqaaiabdMgaPbqabaWccqGGPaqkaaaaaaaa@4833@

where *p*_*ni *_is the probability that person *n *will answer item *i *correctly [or be able to do the task specified by that item], *θ *is person ability, and *b *is the item difficulty parameter.

From this, the expected pattern of responses to an item set is determined given the estimated *θ*. and b. When the observed response pattern coincides with, or does not deviate greatly from, the expected response pattern, the items constitute a true Rasch scale [11]. Taken with confirmation of local independence of items – that is, the absence of residual associations in the data after the Rasch trait has been removed – unidimensionality is confirmed. This model has seen extensive use in the development of health outcome measurement in general [11-20] and also in child-related scales [21]. In health outcome measurement person ability translates to the level of trait (for example, disease severity, functional capacity, or QoL) exhibited by the person and item difficulty to the level of disease severity or (for example) functional capacity manifest in the item. According to the Rasch model, an individual with a severe level of disease would be highly likely to affirm (in the case of a dichotomous item) an item that represented a mild level of the disease.

Within the framework of Rasch measurement, cross-cultural validity can be examined. Essentially, the scale should work in the same way irrespective of which cultural group is assessed. Thus, the location of items along the measurement construct should remain the same between countries. This type of analysis is given the name *differential item functioning *(DIF) [22]. An individual's responses to items should not be affected by factors (such as age, gender and culture) external to the scale and the trait being assessed. DIF occurs when one of these external factors means that one group (for example, males) are significantly more or less likely to affirm an item (or score significantly higher on an item) than females. The basis of the DIF approach lies in the item response function, the S-shaped trace of the proportion of individuals at the same level of trait who affirm an item. Under the requirement that the trait under consideration is unidimensional, if the item measures the same trait across groups then, except for random variations, the same curve should be found irrespective of the nature of the group for which a function is plotted. Items that do not yield the same item response function for two or more groups exhibit DIF and violate the requirement of unidimensionality. The statistical test of DIF is an ANOVA of the residuals.

As noted above, identically worded items may have different PRO values in different cultures (a common cause of DIF). Where some but not all items display such DIF, it is possible to treat them as a different item in each country. This is illustrated in Table 1, where Item 1 effectively represents a separate item in each country and is linked by item 2). This procedure is referred to as "splitting" items across countries. The analysis is re-run on the new item sets. Finally, a Principal Components Analysis (PCA) of the residuals is undertaken to confirm the assumption of local independence of items and thus unidimensionality of the scale. The residual is the difference between what is expected and what is observed and represents what remains when the 'Rasch factor' has been extracted from the data. Therefore, the first factor of the PCA is the primary contributor to the variance of the data when this Rasch factor has been discounted. If the first factor of the PCA accounts for less than 30% of the variance, we can conclude that the scale is undimensional [23].

**Table 1:** Example of adjustment for DIF by splitting item across countries

Country	Item 1a	Item 1b	Item 1c	Item 2
A	*			*
B		*		*
C			*	*

Time-points for each participant were included as separate and individual observations for the analysis to ensure that all time points were calibrated onto the same logit scale. This meant that each person was entered into the analysis three or four times, once for each time point. Data from the European and US trials were calibrated separately. Such an approach is essential when determining change in score on a PRO over time.

Chi^2 ^statistics were used to evaluate individual item and overall scale fit to the Rasch model. Significant Chi^2 ^statistics are indicative of inadequate fit to the model. Due to the number of statistical tests undertaken, Bonferroni corrections were applied to fit analyses [24]. The Rasch analysis employed RUMM 2010 software [25].

### 2. Country-specific validation studies

Following identification of unidimensional CIAD scales it was necessary to analyze the validation study results from each country to determine whether the measures were reliable and valid. Separate studies were conducted in each country (in parallel with the trials) to determine the psychometric properties of the new measure. Details of the samples are shown in Table 2.

**Table 2:** Demographic information for the trial samples and the validation studies

	**European trial**	**US trial**
**N**	208	243
**German (%)**	87 (41.8)	
**French (%)**	52 (25.0)	
**Dutch (%)**	48 (23.1)	
**English (%)**	21 (10.1)	
**American (%)**		243 (100)
**Male (%)**	53.4	51.9
**Mean (SD) age – years**	4.7 (2.5)	4.0 (1.8)

#### Reliability

The reliability of the CIAD was assessed using the test-retest method. Where an instrument is required for use in a clinical trial or for monitoring individual patients, a high reliability is desirable (that is, a correlation coefficient of at least 0.85) [26].

#### Internal consistency

Internal consistency was assessed using Cronbach's alpha coefficients. Values below 0.70 are indicative of individual items not contributing adequately to the overall scale [27].

#### Validity

Construct validity was tested by assessing known groups validity. Scores on the CIAD were related to perceived severity of disease, whether or not the child was experiencing a flare-up and whether or not the face or hands were affected. Differences in QoL scores between the assessment groups were tested by Mann-Whitney U Test where there were two independent groups or Kruskal-Wallis One-way Analysis of Variance where there were three or more independent groups.

### 3. Application of the CIAD in the clinical trials

All trial analyses were carried out on an intention to treat (ITT) data set, using last observation carried forward (for total score – not for individual item missing data).

A simple ANOVA model was employed to examine baseline differences in QoL scores. Analysis of Covariance models were used to test for differences in change scores. The analyses were performed on the logit person estimates obtained from the Rasch analyses, which were then transformed into integers on a 0–100 scale.

An α-level of 0.05 was adopted.

## Results

### Identification of the CIAD for the European trial

Working with the 12 items that were specific to the child, the scale was initially analyzed separately for the four countries (France, Germany, The Netherlands and the UK). Overall fit of the data to the Rasch model ranged from acceptable to good within these countries. Germany and France each had one item that displayed misfit.

When the data were pooled across countries, initial overall fit of the data to the model was poor and three items; '*Other children won't play with her'*, '*She can't join in activities with other children'*, and '*She sleeps badly most nights'*, were found to misfit.

Analysis by country revealed four items that displayed significant DIF, displaying a differential impact of AD across these countries. These items were; '*She whinges all the time'*, '*She is very moody'*, '*She often gets angry with me' *and '*She cannot be comforted'*. Post hoc analysis (Tukey's Test) on these items revealed that the UK and the Netherlands were the countries that displayed most significant difference on these items. Consequently, these four items were each split into three separate items; one for the UK alone, one for the Netherlands alone and one for Germany and France together.

This produced a 20-item scale (eight link/original items and 12 split items). Analysis of this scale revealed that the same three items misfit as with the original 12-item scale. Removal of these items produced good overall fit of the data to the Rasch model, Chi^2 ^= 117.88, df = 75, p < 0.002.(see Table 3)

**Table 3:** CIAD solutions for Europe and the US

**Item**	**Europe**			**US**
	
	**Item deleted**	**Item split by country**	**Link item**	**Item deleted**
Other children won't play with him.				
He can't join in activities with other children.				
He sleeps badly most nights.				
He whinges all the time.				
He is very moody.				
He often gets angry with me.				
He cannot be comforted.				
Other children don't like holding his hand.				
He is often irritable.				
He misses out on a lot of childhood activities.				
He is very demanding.				
He is often restless.				

Principal components analysis (PCA) of the residuals indicated that the first residual factor explained 16% of the variance. As only a small proportion of the variance was explained both the assumption of local independence and unidimensionality were confirmed.

### Identification of the CIAD for the US trial

Initial fit of the 12 items was poor and five items had to be deleted before a good fit to the model was found. These items were; '*Other children don't like holding her hand'*, '*She can't join in activities with other children'*, '*She is often irritable'*, '*She whinges all the time' *and '*She sleeps badly most nights'*. Following the removal of these items, overall fit to the model was good: Chi^2 ^= 42.58, df = 28, p < 0.004. Once again the PCA of the residuals explained little (21%) of the variance.

### Traditional psychometric properties of the CIAD

Having identified the item sets for the CIAD in Europe and the US the validation data from each country were analyzed to ensure that the new scale had adequate psychometric properties in addition to unidimensionality and freedom from DIF. Reproducibility (test-retest reliability) was generally good ranging from 0.78 in the Netherlands to 0.86 in the US. Only 22 Dutch parents completed questionnaires on both occasions, increasing the uncertainty of this finding. Internal consistency was adequate on both occasions in all countries, with alpha coefficients ranging from 0.72 to 0.85. Scores on the CIAD correlated between 0.38 and 0.65 with those on the Psychological General Well-Being Index, indicating that the scales, while related, measure different constructs. Finally, scores on the measures were related, as expected, to perceived severity of disease (assessed by a single global item of current disease severity), whether or not a flare-up was being experienced and whether or not the face or hands were affected in all countries except Germany. The sample in Germany was less severe than that in the other countries and this lack of variability in scores may account for the failure to obtain statistically significant differences. Overall it can be concluded that the different language versions of the CIAD are valid and reliable, despite the relatively low number of items included in the scale.

### Application of the CIAD in the clinical trials

Demographic information for the ITT datasets in the European and US studies are shown in Table 2. T-tests of age and Chi^2 ^tests of gender conducted at baseline revealed no significant difference by treatment group in either study. Similarly, no difference in country by treatment group was found in the European study.

**Figure 1:**
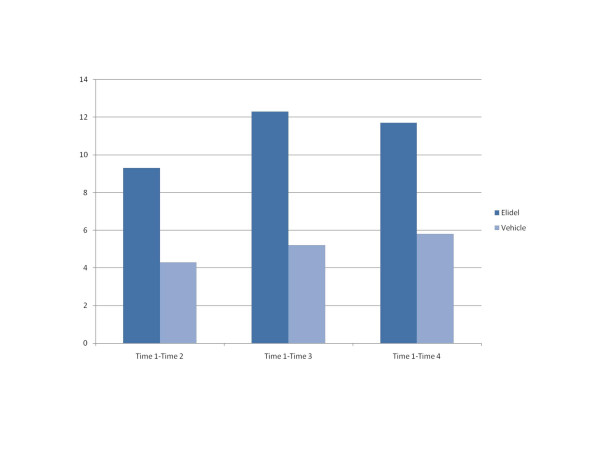
CIAD change scores in European trial.

CIAD scores in European and US trials are shown in Table 4. Change in scores for the Elidel and placebo groups are illustrated in Figures 1 (Europe) and 2 (US). These show that the improvement in CIAD scores for the Elidel group are more marked than those in the placebo group in both trials. The outcome statistics (Table 5) indicate that these differences were only statistically significant for the European trial.

**Table 4:** Descriptive scores for the CIAD

		**Baseline**	**Time 2**	**Time 3**	**Time 4**
***European trial***					
**Total sample**	**N**	208	208	208	208
	**mean**	32.3	24.6	22.3	22.5
	**SD**	23.7	25.0	23.7	24.0
**Elidel**	**N**	140	140	140	140
	**mean**	32.3	23.0	20.0	20.6
	**SD**	23.4	25.0	23.4	24.0
**Conventional**	**N**	68	68	68	68
Therapy	**mean**	32.3	28.0	27.1	26.5
	**SD**	24.9	24.7	23.9	23.7
***US trial***					
**Total sample**	**N**	243	243	243	
	**mean**	23.9	19.9	19.1	
	**SD**	23.6	22.5	22.2	
**Elidel**	**N**	159	159	159	
	**mean**	24.4	19.6	18.1	
	**SD**	23.2	22.0	21.1	
**Vehicle**	**N**	84	84	84	
	**mean**	22.9	20.5	20.8	
	**SD**	24.5	23.7	24.0	

**Table 5:** Outcome Statistics for CIAD

	**F value**	**Df**	**P**	**Mean change**	
				**Elidel**	**Placebo**

**European study**					
**Time 1-Time 2**	3.38	1, 205	0.055	-9.3	-4.3
**Time 1-Time 3**	7.20	1, 205	0.008	-12.3	-5.2
**Time 1-Time 4**	4.53	1, 205	0.035	-11.7	-5.8
**US studies**					
**Time 1-Time 2**	0.88	1, 240	0.348	-4.9	-2.4
**Time 1-Time 3**	2.43	1, 240	0.120	-6.3	-2.1

**Figure 2:**
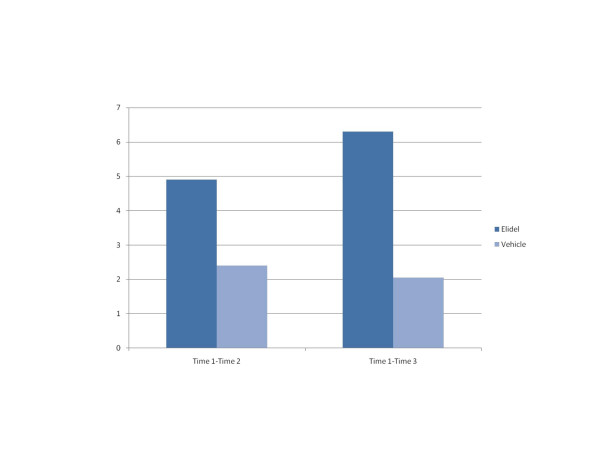
CIAD change scores in US trial.

The standard deviations of baseline CIAD scores were relatively high indicating that the impact of AD on children varies considerably. Furthermore, there was a relatively high drop out rate in the studies (62.2% in the European trial and 40.2% in the US trial). Both factors are likely to have reduced the ability of the CIAD to show differences between treatment arms and suggest the need for larger sample sizes in order to demonstrate the benefits of treatment.

## Discussion

The present study has succeeded in identifying a valid instrument for assessing the impact of AD on children (the CIAD). In order to analyze the clinical trial data in terms of the impact of the treatment on the affected children it was necessary to ensure both that the items fit the measurement model and that there was no DIF associated with cultural differences between countries in the European trial.

Even with careful adaptation of measures major problems can occur where issues are valued differently in different countries. Assuming that all items in a scale fit the measurement model in a particular country no problem would arise until there was a need to combine data from different countries. Such amalgamation of data could lead to treatment differences being masked by DIF. As clinical trials are now frequently multinational, DIF is a major concern that needs to be addressed when interpreting patient-reported outcomes.

Three of the 12 draft CIAD items misfit in Europe and five in the US and these items were excluded from the respective trial analyses. The European CIAD comprised 9 items (3 unique to Europe) while the US measure consisted of 7 items (1 unique to the US) with 6 items being common to both versions. However, three items that misfit in the US were employed in the analysis of the European trial, where they were found to fit. It is not clear why items misfitted in specific countries. However, it is possible that certain translations were not sufficiently precise or that issues were not perceived as problems in some countries. Such issues would be expected to arise when adapting any outcome measure for use in a new language and/or culture.

For the analysis of the European study a novel approach was adopted in which items shown to be valued differently in different countries were treated as individual items. This method of controlling for DIF allowed data from the different countries to be combined in a valid manner. Thus, while all scores were derived from a valid PRO scale, the analyses were based on different item sets in each country. Such an approach relies on the ability to calibrate items from different countries onto the same underlying metric scale, given that some items are common and can provide a linkage across countries. This provides a possible means of overcoming DIF associated with cultural differences in QoL assessment which cannot be avoided where a fixed set of items is employed. Previous instrument development programmes have dealt with such culture-related DIF by rejecting all items exhibiting this problem (see for example, Doward et al, 2003). Allowing items to have different values in each culture means that items considered highly relevant by respondents could be kept in the scales, even where they scale differently in each country – thereby maintaining the face validity of the final scale.

A weakness of the CIAD is its limited number of items. It would be possible to increase the responsiveness of the CIAD by generating additional items and ensuring that the scale excludes misfitting items and others that exhibit DIF across countries. However, such a process is relatively time consuming and expensive.

The CIAD has been shown (like the PIQoL-AD) to fit the Rasch model, providing a metric transformation of ordinal data, and with the absence of significant patterns in the residuals, a unidimensional scale. Use of the two measures in clinical studies would allow the assessment of the impact of AD on both the child and his or her parent, providing a powerful test of the effectiveness of interventions.

## Abbreviations

AD – Atopic dermatitis; CIAD – The Childhood Impact of Atopic Dermatitis; PCA – Principle Components Analysis; PIQoL-AD; The Parents' Index of Quality of Life – Atopic Dermatitis; QoL – quality of Life; PRO – Patient Reported Outcome.

## Competing interests

Three of the authors are employed by Galen Research, an independent research company that has received sponsorship by Novartis pharmaceuticals.

## Authors' contributions

SM: Designed the study, conducted interviews and drafted the manuscript.

LD: Designed the study and conducted interviews and qualitative analysis.

DM: Undertook analyses and prepare the manuscript.

AT: Undertook analysis and reporting.

GL: Undertook analyses and reporting.

JG: Helped design the study and prepare the manuscript.

All authors read and approved the final manuscript.
